# Inflammatory conditions partly impair the mechanically mediated activation of Smad2/3 signaling in articular cartilage

**DOI:** 10.1186/s13075-016-1038-6

**Published:** 2016-06-23

**Authors:** Wojciech Madej, Pieter Buma, Peter van der Kraan

**Affiliations:** Orthopedic Research Laboratory, Radboud University Medical Center, Geert Grooteplein-Zuid 10, route 547, 6525 GA Nijmegen, The Netherlands; Experimental Rheumatology, Radboud University Medical Center, Geert Grooteplein 26-28, route 272, 6525 GA Nijmegen, The Netherlands

**Keywords:** Dynamic mechanical compression, Transforming growth factor beta, Osteoarthritis, Cartilage, Inflammation

## Abstract

**Background:**

Joint trauma, which is frequently related with mechanical overloading of articular cartilage, is a well-established risk for osteoarthritis (OA) development. Additionally, reports show that trauma leads to synovial joint inflammation. In consequence, after joint trauma, cartilage is influenced by deleterious excessive loading combined with the catabolic activity of proinflammatory mediators. Since the activation of TGF-β signaling by loading is considered to be a key regulatory pathway for maintaining cartilage homeostasis, we tested the effect of proinflammatory conditions on mechanically mediated activation of TGF-β/Smad2/3P signaling in cartilage.

**Methods:**

Cartilage explants were subjected to dynamic mechanical compression in the presence of interleukin-1 beta (IL-1β) or osteoarthritic synovium-conditioned medium (OAS-CM). Subsequently, the activation of the Smad2/3P pathway was monitored with QPCR analysis of reporter genes and additionally the expression of receptors activating the Smad2/3P pathway was analyzed. Finally, the ability for mechanically mediated activation of Smad2/3P was tested in human OA cartilage.

**Results:**

IL-1β presence during compression did not impair the upregulation of Smad2/3P reporter genes, however the results were affected by IL-1β-mediated upregulations in unloaded controls. OAS-CM significantly impaired the compression-mediated upregulation of *bSmad7* and *Tgbfb1*. IL-1β suppressed the compression-mediated *bAlk5* upregulation where 12 MPa compression applied in the presence of OAS-CM downregulated the *bTgfbr2*. Mechanically driven upregulation of Smad2/3P reporter genes was present in OA cartilage.

**Conclusions:**

Proinflammatory conditions partly impair the mechanically mediated activation of the protective TGF-β/Smad2/3P pathway. Additionally, the excessive mechanical compression, applied in the presence of proinflammatory conditions diminishes the expression of the type II TGF-β receptor, a receptor critical for maintenance of articular cartilage.

## Background

Mechanical signals have been shown to play a pivotal role in articular cartilage development, tissue maintenance, but also tissue degradation. The mechanical microenvironment acts as a regulator of stem cell fate and leads the chondrogenesis in early embryonic development [1]. Later on, during normal daily activity, cartilage experiences loading within the physiological range of forces [2]. This has been shown to play an essential role in cartilage preservation and in the anabolic production of cartilage matrix molecules [3, 4]. On the other hand, abnormal or excessive cartilage loading, often related with joint trauma and local cartilage mechanical overloading, e.g., as a result of meniscus resection [5], not only leads to direct cartilage damage but can drive the activation of catabolic processes in chondrocytes and cell apoptosis, which leads to further tissue damage [6, 7].

Furthermore, trauma-related damage of articular cartilage and other joint tissues, like meniscus or ligaments, cause the release of tissue debris into joint space contributing to synovial inflammation [8]. Subsequently, inflamed synovium will secrete a number of soluble inflammatory mediators into the joint space, including cytokines and chemokines [9]. Mostly studied are interleukin-1 beta (IL-1β) and tumor necrosis factor alpha (TNF-α), which have both been shown to suppress matrix synthesis and to promote catabolic processes in cartilage [10]. However, also other proinflammatory mediators, like interleukin-6 (IL-6) and interleukin-8 (IL-8), have been identified in synovial fluid of injured joints [9]. In consequence, joint trauma leads to a condition in which articular cartilage is influenced by deleterious excessive loading combined with a catabolic action of proinflammatory mediators.

Transforming growth factor beta (TGF-β)-induced SMAD family member protein (Smad)2/3P signaling has been shown as a crucial signaling in cartilage, since it prevents the deleterious terminal differentiation of cartilage cells [11]. Recently, we have shown that dynamic mechanical compression of healthy articular cartilage is a potent inducer of Smad2/3P signaling in chondrocytes [12]. Nevertheless, data indicate that the effects of cartilage excessive compression with a combination of catabolic action of proinflammatory mediators play a role in development of degenerative joint disease like osteoarthritis (OA) [13, 14]. That is because joint injury is a well-established risk factor for OA [15], and inflammation has been shown to play a key role in OA development in posttraumatic joints [14].

Here, we hypothesized that compression in combination with proinflammatory conditions will be less effective in the induction of Smad2/3P signaling in articular cartilage. Subsequently, a lack of mechanically mediated Smad2/3P would disrupt cartilage maintenance and lead to tissue loss. To test this hypothesis, we investigated the influence of proinflammatory mediators, IL-1β or osteoarthritis-conditioned medium, (OAS-CM) on 3 MPa (physiological) and 12 MPa (excessive) compression-mediated activation of Smad2/3P signaling in healthy bovine articular cartilage. Additionally, we tested if this combination of mechanical and biological factors affects the expression levels of the key TGF-β receptors. Finally, we tested if human osteoarthritic cartilage shows mechanically mediated activation of TGF-β/Smad2/3P.

## Methods

### Articular cartilage explants culture

Metacarpophalangeal joints (MCP) of skeletally mature cows (age range 3–6 years old), obtained from the local abattoir, were processed within 3 hours post-mortem to isolate full-thickness articular cartilage explants. Explants were isolated with use of a 4-mm biopsy punch (Kai-medical, Tokyo, Japan). After isolation, all explants were cultured in Dulbecco’s modified Eagle’s medium: nutrient mixture F-12 (DMEM/F-12) (Gibco®, Paisley, UK) containing Antibiotic-Antimycotic (containing 10,000 units/mL of penicillin, 10,000 μg/mL of streptomycin, and 25 μg/mL of Fungizone®) (Gibco®, Carlsbad, CA, USA). Cartilage explant culture was carried out without serum. Afterward, explants were left for equilibration for 24 hours in standard culture conditions (37 °C, 5 % CO2 and 95 % humidity). The same protocol was used for human OA cartilage explants isolation and culture. Human OA cartilage was obtained from anonymized patients who underwent total knee joint replacement surgery. Explants were isolated from adjacent areas with intact tissue structure (macroscopically inspected).

The study protocol for experiments with animal material obtained from abattoir material did not need animal ethics committee approval.

No consent from patients whose human material was used in this study was needed, because material was obtained from anonymized patients and no personal data was available and needed for our study. Therefore, the study protocol for the experiments with anonymized human material did not need the ethics committee approval.

### Dynamic mechanical compression of articular cartilage explants in the presence of proinflammatory conditions

After 24 hours after isolation, explants were stimulated with hrIL-β1 (1 ng/ml) (R&D Systems, Minneapolis, MN, USA) or with osteoarthritic synovium-conditioned medium OAS-CM 10 % (v/v) [16]). Following 24 hours of culture in proinflammatory conditions (48 hours after explant isolation), explants were randomly assigned to compression groups with corresponding unloaded controls. Compression groups were: 3 MPa compression (physiological stress) and 12 MPa compression (excessive stress). Chosen pressure levels were based on previous evaluations, calculations and published data [12]. Explants from stimulation groups were subjected to force-controlled, sinusoidal, unconfined, dynamic mechanical compression with 3 or 12 MPa pressure and frequency of 1 Hz for 30 minutes (1800 cycles), following the previously published protocol [12]. HrIL β1 (1 ng/ml) or OAS-CM 10 % (v/v) were present in the medium during the compression procedure. After the compression, the loaded articular cartilage and the unloaded control samples were placed back into medium with refreshed hrIL β1 (1 ng/ml) or OAS-CM 10 % (v/v) and back into the culture incubator. At 2 hours after compression, samples were frozen in liquid nitrogen and stored at -80 °C. Additionally, in parallel to the compression experiment, cartilage explants from another group were stimulated with TGF-β1 (10 ng/ml) (Biolegend, San Diego, CA, USA) in combination with hrIL-β1 (1 ng/ml) or OAS-CM 10 % (v/v) for 6 hours (these samples were also pre-incubated with proinflammatory mediators starting at 24 hours before compression).

### Total mRNA isolation and quantitative RT-PCR (QPCR)

A micro-dismembrator (B. Braun Biotech International, Melsungen, Germany) was used to homogenize deep-frozen articular cartilage samples with 1500 RPM for 1 minute. Afterward, using the RNeasy Mini Kit (Qiagen Inc., Valencia, CA, USA) according to the manufacturer’s protocol, total RNA was isolated from homogenate. Isolated RNA was used in reverse transcription reaction to produce complementary DNA (cDNA). Obtained cDNA was used in the QPCR reaction which was carried out with the StepOnePlus Real-Time PCR System (Applied Biosystems, Darmstadt, Germany) according to the manufacturer’s protocol. Primers used are listed in Table 1. Ct values for genes of interest were corrected for the average Ct values of bovine glyceraldehyde 3-phosphate dehydrogenase (*bGapdh*) and bovine ribosomal protein S14 (*bRps14*) to obtain dCt values (in case of bovine material) or they were corrected for the average Ct values of human GAPDH and human ribosomal protein S27 (human RPS27) (in the case of human material).

**Table 1:** Primers used for QPCR

Gene	Full gene name	Ref seq	Product length	Forward 5′-- > 3′	Reverse 5′-- > 3′
*bAlk5*	bovine transforming growth factor, beta receptor 1	NM_174621.2	75	CAGGACCACTGCAATAAAATAGAACTT	TGCCAGTTCAACAGGACCAA
*bGapdh*	bovine glyceraldehyde 3-phosphate dehydrogenase	NM_001034034.2	90	CACCCACGGCAAGTTCAAC	TCTCGCTCCTGGAAGATGGT
*bJunB*	bovine jun B proto-oncogene	NM_001075656.1	139	CCTTCTACCACGACGACTCA	CCGGGTGCTTTGAGATTTCG
*bRps14*	bovine ribosomal protein S14	NM_001077830.2	125	CATCACTGCCCTCCACATCA	TTCCAATCCGCCCAATCTTCA
*bSerpine1*	bovine plasminogen activator inhibitor type 1	NM_174137.2	55	CGAGCCAGGCGGACTTC	TGCGACACGTACAGAAACTCTTGA
*bSmad7*	bovine SMAD family member 7	NM_001192865.1	72	GGGCTTTCAGATTCCCAACTT	CTCCCAGTATGCCACCACG
*bTgfb1*	bovine transforming growth factor, beta 1	NM_001166068.1	80	GGTGGAATACGGCAACAAAATCT	GCTCGGACGTGTTGAAGAAC
*bTgfbr2*	bovine transforming growth factor beta receptor II	NM_001159566.1	141	GGCTGTCTGGAGGAAGAATGA	GTCTCTCCGGACCCCTTTCT
*hALK5*	human transforming growth factor, beta receptor 1	NM_001306210.1	65	CGACGGCGTTACAGTGTTTCT	CCCATCTGTCACACAAGTAAA
*hGAPDH*	human glyceraldehyde 3-phosphate dehydrogenase	NM_001289745.1	143	ATCTTCTTTTGCGTCGCCAG	TTCCCCATGGTGTCTGAGC
*hRPS27*	human ribosomal protein S27	NM_001177413.1	90	TGGCTGTCCTGAAATATTATAAGGT	CCCCAGCACCACATTCATCA
*hSERPINE1*	human plasminogen activator inhibitor type 1	NM_000602.4	213	GTCTGCTGTGCACCATCCCCCATC	TTGTCATCAATCTTGAATCCCATA
*hSMAD7*	human SMAD family member 7	NM_001190821.1	131	CCTTAGCCGACTCTGCGAACTA	CCAGATAATTCGTTCCCCCTGT
*hTGFB1*	human transforming growth factor, beta 1	NM_000660.5	59	GAGGTCACCCGCGTGCTA	TGCTTGAACTTGTCATAGATTTCGTT

### Statistical analysis

**Fig. 1:**
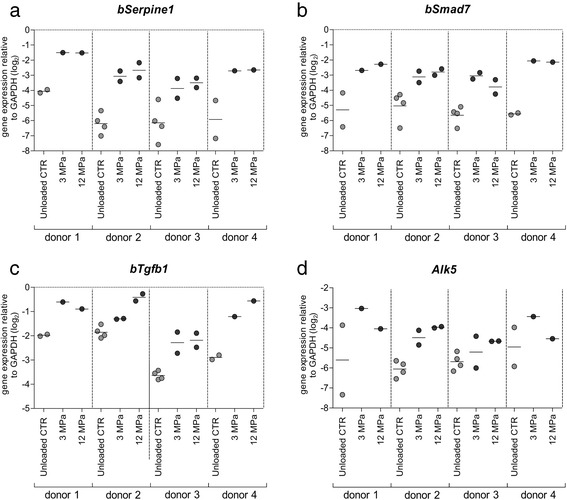
The effect of dynamic mechanical compression of human OA cartilage on the expression of Smad3P-responsive genes. Statistical analysis showed that overall effect of the compression with 3 MPa as well as with 12 MPa of human OA cartilage explants was the induction of the upregulation of *bSerpine1*(**a**), *bSmad7* (**b**), *bTgfb1* (**c**), and *bAlk5* (**d**) in all four different donors. Data are expressed as a grouped column scatter of multiple repeats with displayed mean (each point represents individual experimental repeat of different human OA cartilage explant). ^*^ *p* ≤ 0.05; ^**^ *p* ≤ 0.01; ^***^ *p* ≤ 0.001. *CTR* control, *GAPDH* glyceraldehyde 3-phosphate dehydrogenase

All quantitative data were expressed as a grouped column scatter of multiple repeats with displayed mean. All experiments were repeated four times using cartilage isolated from different animals, *N* = 4 (one cartilage explant sample per condition in the experiment). Because of high variation between patients, in Fig. 1, data of all repeats (individual patients), were presented separately (not pooled).

One-way analysis of variance (ANOVA) with Tukey’s posttest was used to estimate the effect of treatment on gene expression. The univariate ANOVA (with patient as a random factor) was used to test the overall effect of the compression on the gene expression in OA cartilage. The *p* values less than or equal to 0.05 were considered as significant. The statistical analysis was performed with the statistical software package: IBM SPSS 20.0 (IBM Corp., Armonk, NY, USA).

## Results

### The effect of IL-1β on mechanically mediated activation of TGF-β/Smad3P-responsive genes

Previously, we have shown that dynamic mechanical compression can potently induce Smad2/3P signaling activation and TGF-beta1 gene upregulation in articular cartilage [12]. Here, we examined if IL-1β can affect mechanically mediated upregulation of Smad3P-responsive genes (e.g., *Serpine1*, *Smad7*, *and JunB*) and *bTgfb1*.

**Fig. 2:**
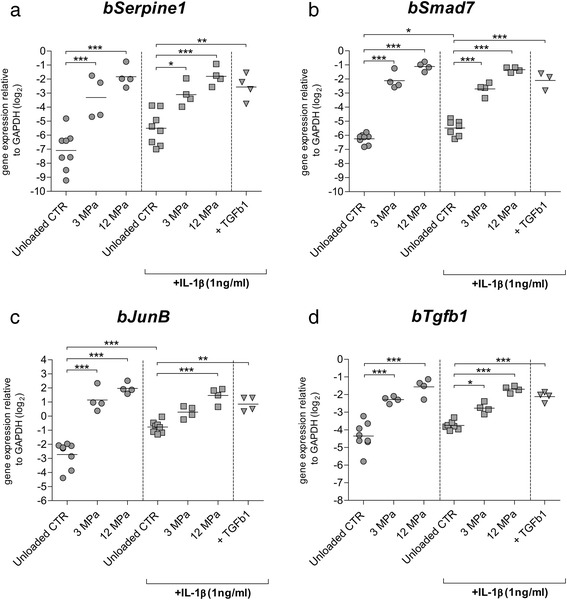
The effect of IL-1β on mechanically mediated activation of Smad3P-responsive genes in bovine articular cartilage. The influence of 3 and 12 MPa dynamic mechanical compression, carried out in absence (*dots*), and presence (*squares*) of IL-1β on relative expression of *bSerpine1* (**a**), *bSmad7* (**b**), *bJunb* (**c**), and *bTgfb1* (**d**). Dynamic mechanical compression with 3 as well as with 12 MPa potently upregulated *bSerpine1*, *bSmad7*, *bJunb*, and *bTgfb1* in conditions without IL-1β. In the presence of IL-1β, mechanically mediated upregulation of Smad3P-responsive genes was also observed. The effect of compression was comparable to the effect of exogenous TGF-β1 stimulation. Data are expressed as a grouped column scatter of multiple repeats with displayed mean (each point represents individual experimental repeat on material isolated from a different animal, *N* = 4). ^*^ *p* ≤ 0.05; ^**^ *p* ≤ 0.01; ^***^ *p* ≤ 0.001. *CTR* control, *GAPDH* glyceraldehyde 3-phosphate dehydrogenase, *IL-1β* interleukin-1 beta, *TGF-β* transforming growth factor beta

A significant induction of *bSerpine1* was observed in both compression groups when compared to unloaded controls (13.7-fold (2^3.8Ct^), *p* < 0.0001 for 3 MPa and 38.2 2^5.3Ct^, *p* < 0.0001 for 12 MPa) (Fig. 2a). In the presence of IL-1β (1 ng/ml), a significant *bSerpine1* upregulation was still noticed in both compressed groups. Compression with 3 MPa induced upregulation of *bSerpine1* by 5.3-fold (2^2.4Ct^) (*p* = 0.03) and compression with 12 MPa induced 13-fold upregulation (2^3.7Ct^) (*p* < 0.0001) when compared to unloaded controls treated with IL-1β (Fig. 2a). Comparable level of upregulation was noticed in cartilage explants stimulated with 10 ng/ml of exogenous rTGF-β1 in a presence of IL-1β (7.7-fold, 2^2.9Ct^) (*p* = 0.005) (Fig. 2a). Analysis of *bSmad7* expression showed a potent and significant upregulation of *Smad7* in both compression groups (17.5-fold, 2^4.1Ct^, *p* < 0.0001 for 3 MPa and 35.1-fold, 2^5.1Ct^, *p* < 0.0001 for 12 MPa) (Fig. 2b). Remarkably, IL-1β treatment stimulated significant upregulation of *bSmad7* in unloaded controls (*p* = 0.044), regardless of loading treatment. Nevertheless, in the presence of IL-1β, the compression-mediated upregulation of *Smad7* was still present when compared to unloaded controls treated with IL-1β (Fig. 2b). 3 MPa compression upregulated *bSmad7* by 6.8-fold (2^2.8Ct^, *p* < 0.0001) whereas 12 MPa compression upregulated it by 17.6-fold (2^4.1Ct^, *p* < 0.0001). The upregulation of *Smad7* caused by compression was similar to the one present in explants stimulated with 10 ng/ml of rTGF-β1 in the presence of IL-1β (10.4-fold, 2^3.4Ct^) (*p* < 0.0001) (Fig. 2b). Further, analysis confirmed that *bJunB* was also potently and significantly upregulated by cartilage compression (Fig. 2c). 3 MPa compression induced a 14.6-fold upregulation of *bJunB* (2^3.9Ct^) (*p* < 0.0001) and the 12 MPa compression caused a 25.8-fold upregulation of this gene (2^4.7Ct^) (*p* < 0.0001). However, a very significant upregulation of *bJunB* (*p* < 0.0001) was also noticed in unloaded controls treated with IL-1β, regardless of loading treatment (Fig. 2c). Because of that, in the presence of IL-1β, a significant *bJunB* upregulation was observed only in 12 MPa compressed cartilage, 4.8-fold, 2^2.2Ct^) (*p* < 0.0001) (Fig. 2c). The level of this upregulation was comparable to the one detected in explants stimulated with 10 ng/ml of rTGF-β1 in the presence of IL-1β (Fig. 2c).

Analysis of the expression of *bTgfb1* demonstrated a significant upregulation of this gene by both levels of compression when compared to the unloaded controls (Fig. 2d). 3 MPa compression induced the expression of *bTgfb1* by 4.2-fold (2^2.1Ct^, *p* < 0.0001) and 12 MPa induced it by 6.9-fold (2^2.^8Ct, *p* < 0.0001) (Fig. 2d). In the presence of IL-1β, a significant induction of *bTgfb1* expression by the compression was also observed (Fig. 2d). 3 MPa compression induced a 2-fold upregulation of *bTgfb1* (2^1.0Ct^) (*p* = 0.033) and 12 MPa induced a 4.2-fold upregulation of this gene (2^2.1Ct^) (*p* < 0.0001) when compared to unloaded controls treated with IL-1β (Fig. 2d). This effect was comparable to the effect of exogenous 10 ng/ml rTGF-β1 stimulation in the presence of IL-1β.

Overall, fold induction by compression in the presence of IL-1β was lower, although not significant, than an absence of IL-1β, but this appears mainly to be caused by elevated basal expression of responsive genes in the presence of IL-1β.

### The effect of OAS-CM on mechanically mediated activation of TGF-β/Smad3P-responsive genes

Subsequently, the effect of 10 % OAS-CM on the loading-mediated regulation of the Smad3P-responsive genes and *bTgfb1* was analyzed.

**Fig. 3:**
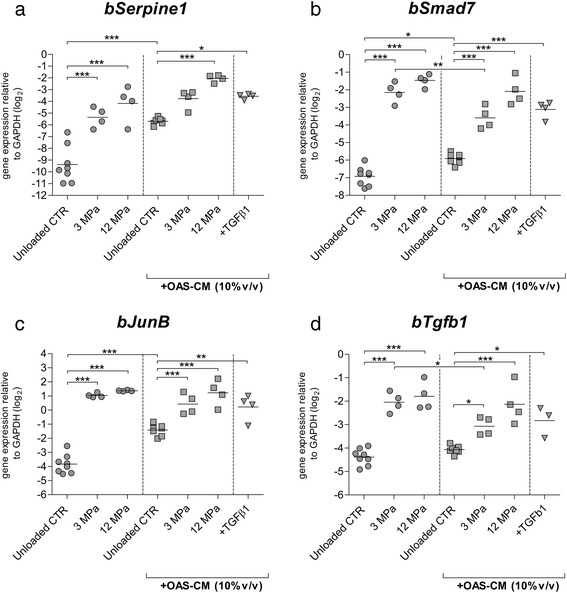
The effect of OAS-CM on mechanically mediated activation of Smad3P-responsive genes in bovine articular cartilage. The influence of 3 and 12 MPa dynamic mechanical compression, carried out with OAS-CM (*squares*) or without (*dots*), on relative expression of *bSerpine1* (**a**), *bSmad7* (**b**), *bJunb* (**c**), and *bTgfb1* (**d**). Dynamic mechanical compression with 3 as well as with 12 MPa potently upregulated *bSerpine1*, *bSmad7*, *bJunb*, and *bTgfb1* in conditions without OAS-CM. In unloaded controls treated with OAS-CM, a significant *bSerpine1*, *bSmad7*, and *bJunb* upregulation was observed. Regardless of this issue, in the presence of OAS-CM a mechanically mediated upregulation of *bSerpine1*, *Smad7*, *JunB*, and *bTgfb1* was noticed. Nevertheless, in the presence of OAS-CM, the 3 MPa compression-mediated upregulation of *bSmad7* and *bTgfb1* was significantly lower than the effect of 3 MPa in conditions without OAS-CM. The effect of compression in the presence of OAS-CM was comparable to the effect of exogenous TGF-β1 stimulation in the presence of OAS-CM. Data are expressed as a grouped column scatter of multiple repeats with displayed mean (each point represents individual experimental repeat on material isolated from a different animal, *N* = 4). ^*^ *p* ≤ 0.05; ^**^ *p* ≤ 0.01; ^***^ *p* ≤ 0.001. *CTR* control, *GAPDH* glyceraldehyde 3-phosphate dehydrogenase, *OAS-CM* osteoarthritic synovium-conditioned medium, TGF-β transforming growth factor beta

As before, mechanical compression with 3 MPa, as well as with 12 MPa, induced significant and potent upregulation of *bSerpine1* (*p* < 0.0001 in both cases; Fig. 3a). Remarkably, addition of 10 % OAS-CM inducted a potent upregulation of *bSerpine1* in unloaded controls, regardless of compression treatment (12.8-fold, 2^3.7Ct^, *p* < 0.0001) (Fig. 3a). Because of these, high levels of *bSerpine1* in unloaded controls, no significant induction of *bSerpine1* was observed in 3 MPa compressed cartilage in the presence of 10 % OAS-CM, however it was observed in 12 MPa compressed cartilage (12.6-fold, 2^3.7Ct^, *p* < 0.0001) (Fig. 3a). Comparable level of *bSerpine1* upregulation was noticed in cartilage stimulated with exogenous rTGF-β1 in the presence of 10 % OAS-CM (Fig. 3a). Analysis of *bSmad7* expression showed potent and significant upregulation in 3 MPa and 12 MPa compressed cartilage (*p* < 0.0001 in both conditions) (Fig. 3b). In the presence of 10 % OAS-CM, a significant upregulation of *bSmad7* was noticed in unloaded controls (*p* = 0.014, 2.0-fold, 2^1.0Ct^) (Fig. 3b). Nevertheless, in the presence of 10 % OAS-CM, the upregulation of *bSmad7* induced by 3 MPa compression was still noticeable. Remarkably, the level of this upregulation was significantly lower (5.5-fold, 2^2.5Ct^, *p* = 0.009) compared to the one in 3 MPa compressed cartilage without OAS-CM (Fig. 3b). In cartilage compressed with 12 MPa in the presence of 10 % OAS-CM the upregulation of *bSmad7* (*p* < 0.0001, 14.1-fold, 2^3.8Ct^) was as potent as the one induced by the 12 MPa compression in the condition without 10 % OAS-CM (Fig. 3b). Comparable levels of *bSmad7* upregulation were noticed in cartilage stimulated with exogenous rTGF-β1 in the presence of 10 % OAS-CM. Analysis of *bJunB* showed analogous results as in the case of *bSerpine1*. Mechanical compression with 3 MPa, as well as with 12 MPa, induced significant and potent upregulation of *bSerpine1* (*p* < 0.0001 in both cases) (Fig. 3c). Also a significant induction of *bJunB* was observed in unloaded controls stimulated with 10 % OAS-CM (5.3-fold, 2^2.4^Ct, *p* < 0.0001) (Fig. 3c). Regardless of this effect, in the presence of 10 % OAS-CM in both compression groups a significant upregulation of *bJunB* was observed (3.6-fold, 2^1.8Ct^, for 3 MPa and 6.3-fold, 2^2.6Ct^, for 12 MPa, *p* < 0.0001 in both cases), and this upregulation was similar to the one present in cartilage stimulated with exogenous 10 ng/ml rTGF-β1 (Fig. 3c).

In conditions without 10 % OAS-CM, in both compression groups, a significant upregulation of *bTgfb1* was found (*p* < 0.0001 in both cases) (Fig. 3d). In the presence of 10 % OAS-CM, 3 MPa also induced *bTgfb1* upregulation (2-fold, 2^1.0Ct^, *p* = 0.040), however this induction was significantly lower (2.5-fold, 2^1.3Ct^, *p* = 0.049) than without OAS-CM (Fig. 3d). In 12 MPa compression groups this difference was not noticeable, as in presence of OAS-CM, the 12 MPa-induced *bTgfb1* upregulation was as potent as the one observed in 12 MPa compressed cartilage without OAS-CM (Fig. 3d). The effect of compression in the presence of 10 % OAS-CM was comparable to the effect of exogenous 10 ng/ml rTGF-β1 stimulation in the same conditions.

Overall, the effect of the OAS-CM on the loading-mediated upregulation of *bSerpine1* and *bJunB* has been disrupted by the strong effect of the OAS-CM on the induction of these genes in unloaded controls. However, when this effect was not observed, a significantly lower loading-induced upregulation of *bSmad7* and *bTgfb1* was noticed in the presence of OAS-CM.

### The effect of inflammatory conditions and mechanical compression on the expression of TGF-β receptors

Response of chondrocytes to TGF-β is determined by the balance of specific receptors for this growth factor (transforming growth factor, beta receptor I [TGFBR1 (ALK-5)] and transforming growth factor, beta receptor II [TGFBR2]). Here we analyzed whether inflammatory conditions are able to influence the loading-regulated expression of TGF-β receptors.

**Fig. 4:**
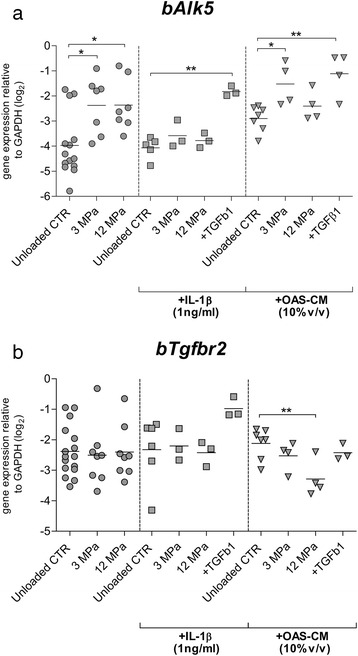
The effect of mechanical compression carried out in presence of inflammatory conditions on the expression of TGF-β receptors. The influence of 3 and 12 MPa dynamic mechanical compression, carried out with IL-1β (*squares*) or with OAS-CM (*triangles*) or without inflammatory conditions (*dots*), on the relative expression of *bAlk5* (**a**) and *bTgfbr2* (**b**). Dynamic mechanical compression with 3 MPa as well as with 12 MPa potently upregulated *bAlk5* in conditions without inflammatory conditions. However, in the presence of IL-1β, 3 MPa as well as 12 MPa mechanical compression had no influence on *bAlk5* expression. In the presence of OAS-CM, only 3 MPa compression upregulated *bAlk5*. Dynamic mechanical compression had no influence on *bTgfbr2* expression in conditions without inflammatory conditions. It had also no influence on *bTgfbr2* expression in the presence of IL-1β. However, in the presence of OAS-CM, 12 MPa compression induced a downregulation of *bTGfbr2*. Data are expressed as a grouped column scatter of multiple repeats with displayed mean (each point represents individual experimental repeat on material isolated from a different animal, *N* = 4). ^*^ *p* ≤ 0.05; ^**^ *p* ≤ 0.01; ^***^ *p* ≤ 0.001. *CTR* control, *GAPDH* glyceraldehyde 3-phosphate dehydrogenase, *IL-1β* interleukin-1 beta, *OAS-CM* osteoarthritic synovium-conditioned medium, *TGF-β* transforming growth factor beta

Analysis of *bAlk5* expression showed that mechanical compression with 3 MPa as well as with 12 MPa could significantly induce expression of this receptor (*p* = 0.018 in both cases; Fig. 4a). Remarkably, IL-1β was able to completely inhibit the mechanically mediated upregulation of *bAlk5* expression induced by 3 MPa as well as by 12 MPa compression (Fig. 4a). This was in contrast with the effect of exogenous TGF-β (10 ng/ml), which even in the presence of IL-1β was able to significantly upregulate *bAlk5* expression (4.7-fold, 2^4.7Ct^, *p* = 0.003). In the presence of 10 % OAS-CM, 3 MPa mechanical compression was still able to upregulate *bAlk5* when compared to unloaded controls (2.58-fold, 2^1.37Ct^, *p* = 0.029). However, in the same conditions, 12 MPa was not able to upregulate *bAlk5* (Fig. 4a). Nevertheless, the upregulation of *bAlk5* induced by exogenous TGF-β was still observed in OAS-CM conditions also (3.4-fold, 2^1.78Ct^, *p* = 0.004).

When *bTgfbr2* expression was analyzed, no effect of compression with 3 MPa as well as with 12 MPa was noticed (Fig. 4b) in the absence of proinflammatory conditions. Also in the presence of IL-1β, no effect of compression was observed. Further, in the presence of 10 % OAS-CM no effect of 3 MPa on *bTgfbr2* was observed. Remarkably, in the presence of OAS-CM, 12 MPa mechanical compression even induced a downregulation of *bTgfbr2* (2.25-fold, 2^1.16Ct^, *p* = 0.010), (Fig. 4b).

### Mechanically mediated activation of TGF-β/Smad3P-responsive genes in human OA cartilage

Analysis of *bSerpine1* expression showed that both mechanical compression levels caused an upregulation of this gene in OA cartilage of all four patients. Statistical analysis showed that, overall the effect of 3 MPa compression was a significant *bSerpine1* upregulation of 6.8-fold (mean upregulation) (2^2.76Ct^, *p* < 0.0001) in all patients and the overall effect of 12 MPa was a significant upregulation of 8.1-fold (2^3.03Ct^, *p* < 0.0001). Subsequent examination of *bSmad7* expression confirmed these results of *bSerpine1* analysis. In OA cartilage of all four patients, an upregulation caused by both mechanical compression regimes was noticed. Statistical analysis showed that the overall effect of 3 MPa compression in all patients was a significant *bSmad7* upregulation for 5.8-fold (mean upregulation) (2^2.52Ct^, *p* < 0.0001) and the effect of 12 MPa was a significant upregulation of 5.4-fold (2^2.44Ct^, *p* < 0.0001). In all four patients an upregulation of *bTgfb1* was also noticed. Statistical analysis showed that the overall effect of 3 MPa compression was a significant upregulation of *bTgfb1* for 2.2-fold (2^1.14Ct^, *p* < 0.0001) whereas in the case of 12 MPa compression, the overall effect was a significant upregulation of this gene of 2.9-fold (2^1.53Ct^, *p* < 0.0001). Analysis of *bAlk5* demonstrated more variation in the effect of the compression. Statistical analysis showed that the overall effect of 3 MPa compression was a significant upregulation of *bAlk5* of 2.5-fold (2^1.36Ct^, *p* < 0.0001) whereas in the case of 12 MPa compression, the overall effect was a significant upregulation of this gene of 2.5-fold (2^1.36Ct^, *p* < 0.0001). Generally, both levels of the compression were able to induce the upregulation of all investigated Smad3P-responsive genes in human OA cartilage.

## Discussion

Previously, we have shown that excessive dynamic mechanical compression alone was not able to induce deleterious alterations of TGF-β signaling in articular cartilage [12]. Of note, we observed that even excessive compression induced a pathway that has been shown to be crucial for cartilage maintenance Smad2/3P signaling [11]. These results were not fully in line with previous observations, which suggested that overloaded cartilage has increased chondrocytes catabolic activity and matrix degradation [6, 17, 18]. However, as we discussed in our previous manuscript, additional proinflammatory conditions present during mechanical compression could alter or impair the mechanically mediated activation of TGF-β signaling in articular cartilage. The rationale for this statement might lay in the data which shows that overloading of the cartilage, that leads to tissue degeneration and OA development, in vivo takes place predominantly after joint trauma [15], where inflammation of the joint is a common fact [9, 19]. That is why, as a follow-up, here we tested if proinflammatory conditions in combination with compression can impair the mechanically mediated activation of TGF-β/Smad2/3P signaling in articular cartilage.

Interplay between proinflammatory mediators and TGF-β signaling has been shown already. van Beuningen et al*.* showed that TGF-β is able to suppress IL-1β-induced proteoglycan degradation in vivo [20, 21]. Furthermore, TGF-β has been shown to counteract IL-1β effect on several levels, not only by downregulation of its receptor but also by upregulation of the IL-1 antagonist IL-1Ra [22, 23]. Separately, compression of articular cartilage has been shown to counteract the IL-1β-induced catabolic activity in chondrocytes [24], which might be associated with mechanically mediated TGF-β action [12, 25]. On the other hand, our analysis showed that IL-1β in the concentration of 1 ng/ml has no significant effect on mechanically mediated activation of Smad2/3P signaling response genes like *Serpine1*, *Smad 7*, or *JunB* in articular cartilage. This showed that IL-1β alone and/or IL-1β-induced signaling in cartilage are not able to interfere with activation of Smad2/3 signaling by compressive load in intact articular cartilage. We observed however, that IL-1β was able to block the mechanically mediated upregulation of Smad2/3P activation receptor (*Alk5*). Considering the fact that mechanically mediated upregulation of *Alk5* is actually depending on the ALK5 receptor [12] and no reduction of ALK5 receptor caused by IL-1β treatment only was observed, it can be concluded that inhibition of mechanically mediated *bAlk5* upregulation by IL-1β is regulated by modulation of intracellular pathways.

The canonical intracellular TGF-β signaling pathway involves phosphorylation of Smad2/3 followed by subsequent formation of a complex with Smad4, the common-Smad. Subsequently, this complex translocates to the nucleus where it binds DNA and regulates gene transcription [26]. On the other hand, in chondrocytes IL-1β signals mainly by activation of nuclear factor kappa B (NF-kB) signaling, a well-validated major catabolic pathway in cartilage degradation [27, 28]. In fact, intracellular interaction of TGF-β-induced Smad signaling and IL-1β-induced signaling has already been shown [29]. Roman-Blas et al*.* showed that IL-1β-induced NF-kB signaling is able to reduce DNA-binding activity of Smad3/4- the main TGF-β signaling-induced gene-regulating complex in adult chondrocytes [30]. However, the lack of significant influence of IL-1β on mechanically mediated induction of *Serpine1*, *Smad 7*, or *JunB*, the Smad3P reporter genes that should also be regulated by Smad3/4 complex [31–33], appears in contrast with the results of Roman-Blas et al. Nevertheless, in results published by Roman-Blas et al*.,* the most prominent effects of IL-1β on DNA-binding activity of Smad3/4 were observed in human OA chondrocytes and not in healthy bovine cartilage cells [29]. Moreover, results published by Roman-Blas et al*.* were observed in the isolated monolayer cells but not in the intact cartilage explants like we used. As it has been shown, chondrocyte isolation for later monolayer culture has an effect on catabolic intracellular signaling pathways [34], including NF-kB signaling [35], which might explain the differences in observations.

Nevertheless, inflamed synovium produces more proinflammatory cytokines than only IL-1β and, as shown by Heldens et al., the catabolic effects of mediators produced by inflamed synovium extend beyond the effect of IL-1β only [36]. Because of that, we tested if OA synovium-conditioned medium, containing multiple proinflammatory mediators [9] can have an impact on the mechanically mediated activation of the Smad2/3P pathway in cartilage. Our data showed that physiological as well as excessive mechanical compression is able to induce the upregulation of Smad3P-responsive genes (*bSerpine1*, *bJunB*) also when applied in the presence of OAS-CM. Nevertheless, we also noticed a very prominent effect of OAS-CM in unloaded cartilage, which upregulated the expression of S*erpine1 and JunB* regardless of the loading treatment, which was most likely caused by soluble factors contained in OAS-CM [37].

This effect was not observed in case of *Smad7* and *Tgfb1* expression. Stable expression levels of *Smad7* and *Tgfb1* allowed us to notice that inflammatory mediators contained in OAS-CM significantly impaired the activation of these genes by physiological compression. One of the possible explanations for this particular response might be explained by the action of TGF-β-activated kinase 1 (TAK1). TAK1 has been identified as a TGF-β/BMP activated intracellular component of mitogen-activated protein kinases (MAPK) pathways [38]. However, TAK1 has been also shown to be a central intracellular kinase for a number of important inflammatory cytokines [39]. Hoffmann et al*.* has shown that TAK1 plays an essential role in Smad2/3P signaling modulation. They demonstrated that overexpression of TAK1 or activation of TAK1 leads to accumulation of all activated Smad2/3 in the cell cytoplasm with their parallel depletion from the nucleus [40], which would impair gene regulation by Smad3 and stay in line with our results. These results might explain why in the presence of inflammatory mediators present in OAS-CM, the loading-mediated upregulation of TGF-β-responsive genes are impaired.

Analysis of the expression levels of the TGF-β/Smad2/3P-responsive genes did not fully confirm our hypothesis that combination of proinflammatory conditions with excessive loading could alter the TGF-β signaling in cartilage. All analyzed TGF-β-responsive genes were upregulated by excessive mechanical compression applied in the presence of either IL-1β or OAS-CM, however some to a significantly lesser extent, demonstrating the inhibitory effect of the proinflammatory conditions on the effect of the physiological compression. Moreover, human OA cartilage, which is known to be exposed to proinflammatory conditions for a prolonged period [41], still demonstrated induction of Smad2/3 genes. However, we cannot conclude anything about the induction level since healthy human cartilage was not available for comparison.

Nevertheless, most importantly, the analysis of the *bTgfbr2* expression revealed a prominent downregulation of the *bTgfbr2* by excessive compression only when applied in the presence of OAS-CM. TGF-β type II receptor (TGFBR2) is the receptor that directly binds TGF-β ligand, which induces the recruitment and subsequent phosphorylation of the type I receptor, thereafter followed by further Smad signaling. It has been shown that TGFBR2 is absolutely critical for maintenance of articular cartilage and loss of this receptor results in total loss of responsiveness to TGF-β, which drives chondrocyte terminal differentiation and development of OA [42]. Baugé et al*.* showed that proinflammatory mediators like IL-1β can reduce *Tgfbr2* expression in OA monolayer chondrocytes [43]. We did not observe a similar effect of proinflammatory conditions alone, but this might be due to the different sensitivity of OA monolayer chondrocytes (used by Baugé et al.) for the used cytokines than healthy cartilage explants [44]. Here we observed the downregulation of *Tgfbr2* at 2 hours after the compression. This time point is too early to see the functional consequences of *Tgfbr2* gene downregulation on TGF-β-responsive gene expression. In the future, it is important to check if the downregulation of the *Tgfbr2* caused by excessive mechanical compression applied in the presence of inflammatory conditions will result in reduced protein expression, if this loss of TGFBR2 is temporary or permanent, and most importantly, if and what are the physiological consequences of this loss.

The major limitation of our study is fact that our conclusions were based only on gene expression data. However, this was mainly driven by the fact that our experiments were performed on the model of intact articular cartilage explants, where cellular proteins are only 0.01 to 0.1 % of the entire tissue volume. High amounts of big ECM protein make the detection of specific membrane or phosphorylated proteins extremely difficult and reproducibility of the result at the protein level is poor.

## Conclusions

Joint trauma is a well-established risk for the development of OA and this is often attributed to the fact that after joint injury the cartilage is overloaded in the presence of inflammatory mediators. However, in this manuscript we point out that excessive mechanical compression with a combination of proinflammatory conditions partly suppresses the mechanically mediated TGF-β/Smad3/2P signaling. Our observations suggest that in the presence of inflammatory conditions, compression is less able to effectively induce the TGF-β/Smad3/2P signaling. Moreover, in our view the most important observation of this study is that excessive compression applied in the presence of inflammatory factors causes a downregulation of the crucial TGF-β receptor TGFBR2. We hypothesize that the loss of TGFBR2 might explain how overloading will induce cartilage damage that transcends its purely mechanical effects. Additionally, this may also indicate why the surgical restabilization of the joint does not reduce the risk of progressive joint degeneration after joint trauma [45].

### Statement

The human material used was completely anonymous surgery surplus material. Patients of the Radboudumc, Nijmegen, The Netherlands are informed about the potential anonymous use of this material and can decline the anonymous use of their material for research. According to Dutch law informed consent is not necessary.

## Abbreviations

ANOVA, analysis of variance; cDNA, complementary DNA; DMEM/F-12, Dulbecco’s modified Eagle’s medium: nutrient mixture F-12; GAPDH, glyceraldehyde 3-phosphate dehydrogenase; IL-1β, interleukin-1 beta; IL-6, interleukin-6; IL-8, interleukin-8; MAPK, mitogen-activated protein kinases; MCP joint, metacarpophalangeal joints; NF-kB, nuclear factor kappa-light-chain-enhancer of activated B cells; OA, osteoarthritis; OAS-CM, osteoarthritic synovium-conditioned medium; QPCR, quantitative real-time polymerase chain reaction; Smad, SMAD family member protein; TAK-1, transforming growth factor beta-activated kinase 1; TGFBR1 (ALK-5), transforming growth factor, beta receptor I; TGFBR2, transforming growth factor, beta receptor II; TGF-β, transforming growth factor beta; TNF-α, tumor necrosis factor alpha

## References

[1] Roddy KA, Prendergast PJ, Murphy P (2011). Mechanical influences on morphogenesis of the knee joint revealed through morphological, molecular and computational analysis of immobilised embryos. PLoS One.

[2] Ahmed AM, Burke DL (1983). In-vitro measurement of static pressure distribution in synovial joints--Part I: Tibial surface of the knee. J Biomech Eng.

[3] Vanwanseele B, Eckstein F, Knecht H, Stussi E, Spaepen A (2002). Knee cartilage of spinal cord-injured patients displays progressive thinning in the absence of normal joint loading and movement. Arthritis Rheum.

[4] Ikenoue T, Trindade MC, Lee MS, Lin EY, Schurman DJ, Goodman SB (2003). Mechanoregulation of human articular chondrocyte aggrecan and type II collagen expression by intermittent hydrostatic pressure in vitro. J Orthop Res.

[5] Baratz ME, Fu FH, Mengato R (1986). Meniscal tears: the effect of meniscectomy and of repair on intraarticular contact areas and stress in the human knee. A preliminary report. Am J Sports Med.

[6] Lin PM, Chen CT, Torzilli PA (2004). Increased stromelysin-1 (MMP-3), proteoglycan degradation (3B3- and 7D4) and collagen damage in cyclically load-injured articular cartilage. Osteoarthritis Cartilage.

[7] Loening AM, James IE, Levenston ME, Badger AM, Frank EH, Kurz B (2000). Injurious mechanical compression of bovine articular cartilage induces chondrocyte apoptosis. Arch Biochem Biophys.

[8] Scanzello CR, McKeon B, Swaim BH, DiCarlo E, Asomugha EU, Kanda V (2011). Synovial inflammation in patients undergoing arthroscopic meniscectomy: molecular characterization and relationship to symptoms. Arthritis Rheum.

[9] Sward P, Frobell R, Englund M, Roos H, Struglics A (2012). Cartilage and bone markers and inflammatory cytokines are increased in synovial fluid in the acute phase of knee injury (hemarthrosis)--a cross-sectional analysis. Osteoarthritis Cartilage.

[10] Goldring MB, Marcu KB (2009). Cartilage homeostasis in health and rheumatic diseases. Arthritis Res Ther.

[11] Yang X, Chen L, Xu X, Li C, Huang C, Deng CX (2001). TGF-beta/Smad3 signals repress chondrocyte hypertrophic differentiation and are required for maintaining articular cartilage. J Cell Biol.

[12] Madej W, van Caam A, Blaney Davidson EN, van der Kraan PM, Buma P (2014). Physiological and excessive mechanical compression of articular cartilage activates Smad2/3P signaling. Osteoarthritis Cartilage.

[13] Gelber AC, Hochberg MC, Mead LA, Wang NY, Wigley FM, Klag MJ (2000). Joint injury in young adults and risk for subsequent knee and hip osteoarthritis. Ann Intern Med.

[14] Lieberthal J, Sambamurthy N, Scanzello CR (2015). Inflammation in joint injury and post-traumatic osteoarthritis. Osteoarthritis Cartilage.

[15] Brown TD, Johnston RC, Saltzman CL, Marsh JL, Buckwalter JA (2006). Posttraumatic osteoarthritis: a first estimate of incidence, prevalence, and burden of disease. J Orthop Trauma.

[16] van Beuningen HM, de Vries-van Melle ML, Vitters EL, Schreurs W, van den Berg WB, van Osch GJ (2014). Inhibition of TAK1 and/or JAK can rescue impaired chondrogenic differentiation of human mesenchymal stem cells in osteoarthritis-like conditions. Tissue Eng Part A.

[17] Stevens AL, Wishnok JS, White FM, Grodzinsky AJ, Tannenbaum SR (2009). Mechanical injury and cytokines cause loss of cartilage integrity and upregulate proteins associated with catabolism, immunity, inflammation, and repair. Mol Cell Proteomics.

[18] Nishimuta JF, Levenston ME (2012). Response of cartilage and meniscus tissue explants to in vitro compressive overload. Osteoarthritis Cartilage.

[19] Bigoni M, Sacerdote P, Turati M, Franchi S, Gandolla M, Gaddi D (2013). Acute and late changes in intraarticular cytokine levels following anterior cruciate ligament injury. J Orthop Res.

[20] van Beuningen HM, van der Kraan PM, Arntz OJ, van den Berg WB (1994). In vivo protection against interleukin-1-induced articular cartilage damage by transforming growth factor-beta 1: age-related differences. Ann Rheum Dis.

[21] van Beuningen HM, van der Kraan PM, Arntz OJ, van den Berg WB (1993). Protection from interleukin 1 induced destruction of articular cartilage by transforming growth factor beta: studies in anatomically intact cartilage in vitro and in vivo. Ann Rheum Dis.

[22] Redini F, Mauviel A, Pronost S, Loyau G, Pujol JP (1993). Transforming growth factor beta exerts opposite effects from interleukin-1 beta on cultured rabbit articular chondrocytes through reduction of interleukin-1 receptor expression. Arthritis Rheum.

[23] Bodo M, Carinci P, Baroni T, Bellucci C, Giammarioli M, Pezzetti F (1998). Role of growth factors on extracellular matrix production by chick embryo fibroblasts in vitro. Antagonist effect of TGF-beta through the control of IL-1 and IL-1Ra secretion. Cytokine.

[24] Chowdhury TT, Bader DL, Lee DA (2006). Dynamic compression counteracts IL-1beta induced iNOS and COX-2 activity by human chondrocytes cultured in agarose constructs. Biorheology.

[25] Bougault C, Aubert-Foucher E, Paumier A, Perrier-Groult E, Huot L, Hot D (2012). Dynamic compression of chondrocyte-agarose constructs reveals new candidate mechanosensitive genes. PLoS One.

[26] Li TF, O’Keefe RJ, Chen D (2005). TGF-beta signaling in chondrocytes. Front Biosci..

[27] Firestein GS, Manning AM (1999). Signal transduction and transcription factors in rheumatic disease. Arthritis Rheum.

[28] Fan Z, Yang H, Bau B, Soder S, Aigner T (2006). Role of mitogen-activated protein kinases and NFkappaB on IL-1beta-induced effects on collagen type II, MMP-1 and 13 mRNA expression in normal articular human chondrocytes. Rheumatol Int.

[29] Roman-Blas JA, Stokes DG, Jimenez SA (2007). Modulation of TGF-beta signaling by proinflammatory cytokines in articular chondrocytes. Osteoarthritis Cartilage.

[30] Yang YC, Piek E, Zavadil J, Liang D, Xie D, Heyer J (2003). Hierarchical model of gene regulation by transforming growth factor beta. Proc Natl Acad Sci U S A.

[31] Dennler S, Itoh S, Vivien D, ten Dijke P, Huet S, Gauthier JM (1998). Direct binding of Smad3 and Smad4 to critical TGF beta-inducible elements in the promoter of human plasminogen activator inhibitor-type 1 gene. EMBO J.

[32] Jonk LJ, Itoh S, Heldin CH, ten Dijke P, Kruijer W (1998). Identification and functional characterization of a Smad binding element (SBE) in the JunB promoter that acts as a transforming growth factor-beta, activin, and bone morphogenetic protein-inducible enhancer. J Biol Chem.

[33] Nagarajan RP, Zhang J, Li W, Chen Y (1999). Regulation of Smad7 promoter by direct association with Smad3 and Smad4. J Biol Chem.

[34] Klatt AR, Paul-Klausch B, Klinger G, Kuhn G, Renno JH, Banerjee M (2009). A critical role for collagen II in cartilage matrix degradation: collagen II induces pro-inflammatory cytokines and MMPs in primary human chondrocytes. J Orthop Res.

[35] Pulai JI, Chen H, Im HJ, Kumar S, Hanning C, Hegde PS (2005). NF-kappa B mediates the stimulation of cytokine and chemokine expression by human articular chondrocytes in response to fibronectin fragments. J Immunol.

[36] Heldens GT, Blaney Davidson EN, Vitters EL, Schreurs BW, Piek E, van den Berg WB (2012). Catabolic factors and osteoarthritis-conditioned medium inhibit chondrogenesis of human mesenchymal stem cells. Tissue Eng Part A.

[37] Schlaak JF, Pfers I, Meyer Zum Buschenfelde KH, Marker-Hermann E (1996). Different cytokine profiles in the synovial fluid of patients with osteoarthritis, rheumatoid arthritis and seronegative spondylarthropathies. Clin Exp Rheumatol.

[38] Yamaguchi K, Shirakabe K, Shibuya H, Irie K, Oishi I, Ueno N (1995). Identification of a member of the MAPKKK family as a potential mediator of TGF-beta signal transduction. Science.

[39] Sakurai H (2012). Targeting of TAK1 in inflammatory disorders and cancer. Trends Pharmacol Sci.

[40] Hoffmann A, Preobrazhenska O, Wodarczyk C, Medler Y, Winkel A, Shahab S (2005). Transforming growth factor-beta-activated kinase-1 (TAK1), a MAP3K, interacts with Smad proteins and interferes with osteogenesis in murine mesenchymal progenitors. J Biol Chem.

[41] Sokolove J, Lepus CM (2013). Role of inflammation in the pathogenesis of osteoarthritis: latest findings and interpretations. Ther Adv Musculoskelet Dis.

[42] Serra R, Johnson M, Filvaroff EH, LaBorde J, Sheehan DM, Derynck R (1997). Expression of a truncated, kinase-defective TGF-beta type II receptor in mouse skeletal tissue promotes terminal chondrocyte differentiation and osteoarthritis. J Cell Biol.

[43] Baugée C, Legendre F, Leclercq S, Elissalde JM, Pujol JP, Galera P (2007). Interleukin-1beta impairment of transforming growth factor beta1 signaling by down-regulation of transforming growth factor beta receptor type II and up-regulation of Smad7 in human articular chondrocytes. Arthritis Rheum.

[44] Guicheux J, Palmer G, Relic B, Mezin F, Caverzasio J, Apostolides P (2002). Primary human articular chondrocytes, dedifferentiated chondrocytes, and synoviocytes exhibit differential responsiveness to interleukin-4: correlation with the expression pattern of the common receptor gamma chain. J Cell Physiol.

[45] Chalmers PN, Mall NA, Moric M, Sherman SL, Paletta GP, Cole BJ (2014). Does ACL reconstruction alter natural history?: A systematic literature review of long-term outcomes. J Bone Joint Surg Am.

